# Medical Management of a Case of Inferior-Posterior Wall ST Elevation Myocardial Infarction Complicated by Pseudoaneurysm

**DOI:** 10.7759/cureus.7675

**Published:** 2020-04-15

**Authors:** Olusola Adekoya, Obinna Mmagu, Nathaniel Dittoe, Thomas Ruff

**Affiliations:** 1 Internal Medicine, Kettering Medical Center, Dayton, USA; 2 Cardiology, Kettering Health Network, Dayton, USA

**Keywords:** pseudoaneurysm, thrombosed pseudoaneurysm, inferior-posterior myocardial infarction

## Abstract

Left ventricular pseudoaneurysm is a rare complication associated with high morbidity and mortality when symptomatic and is usually managed aggressively to prevent or reduce risk of mortality. Herein, we present the case of a 73-year-old man who underwent coronary artery bypass grafting 10 years ago, now presenting with an inferior-posterior wall myocardial infarction complicated by pseudoaneurysm. This case highlights the need for individual clinical assessment of patient presentation with consideration for medical management in acute settings in patients with pseudoaneurysm of the left ventricle.

Despite the natural history of rare complications with pseudoaneurysms such as rupture and potentially fatal cardiac tamponade, infections, and arrhythmias, it is important to note that pseudoaneurysms can present with coronary artery disease and congestive heart failure, and clinicians need to have an index of suspicion for pseudoaneurysms despite advancement in medical and interventional management of coronary artery disease.

## Introduction

Left ventricular pseudoaneurysm is a rare complication due to improved medical and interventional management of acute myocardial infarction (MI) [1]. It can be associated with high morbidity and mortality when symptomatic, and is usually managed aggressively to prevent or reduce the risk of mortality. The incidence of pseudoaneuryms is reportedly as low as <0.3% [1,2]. When they occur, mortality can be as high as 40% with complications such as rupture and potentially fatal cardiac tamponade, thromboembolism, focus of infection, and arrhythmia [3]. Despite the natural history of rare complications with pseudoaneurysms, it is important to note that pseudoaneurysms can present with coronary artery disease and congestive heart failure, and clinicians need to have an index of suspicion for pseudoaneurysms despite advancement in medical and interventional management of coronary artery disease [2]. This will help reduce morbidity and mortality in these patients.

## Case presentation

A 73-year-old male presented with acute-onset substernal chest pain which had been preceded by bilateral arm pain for two days prior to presentation to the emergency department. He described the pain as moderate in intensity, radiating to his back and associated with shortness of breath, nausea, and diaphoresis. He was brought in by the emergency medical service and received aspirin prior to arrival.

His medical history included hypertension, renal cell carcinoma, and previous anterior wall MI. He underwent coronary artery bypass grafting (CABG) in 2009 for severe coronary three-vessel disease: 100% left anterior descending (LAD), 99% left circumflex, and 75% right coronary artery (RCA) stenotic disease. He subsequently underwent revascularization with left internal mammary artery to LAD, saphenous vein graft in the first obtuse marginal artery (SVG-OM1), and SVG to posterior descending coronary artery. He was noncompliant with medications and continued to use tobacco and alcohol regularly. Intermittently, he took aspirin 81 mg and was lost to cardiology follow-up.

**Figure 1:**
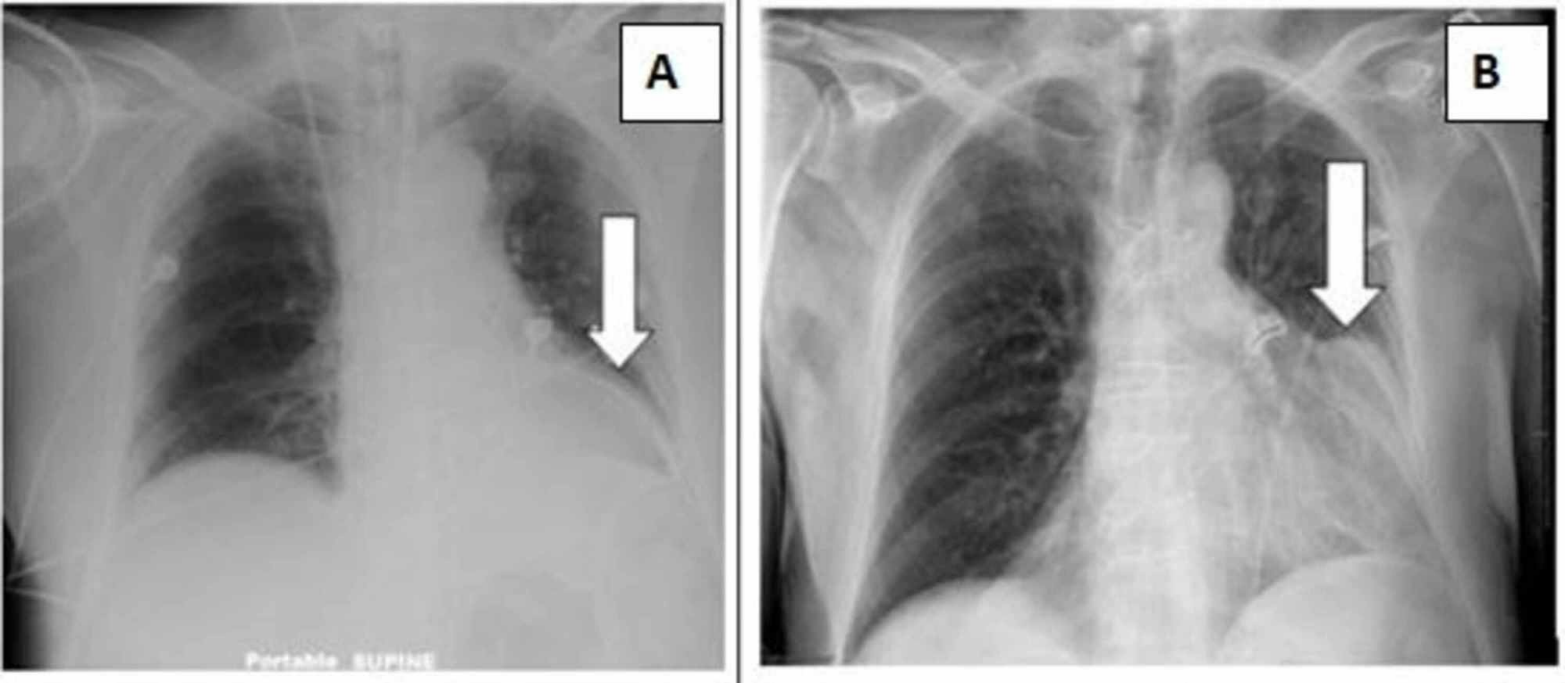
Chest X-ray during current hospitalization (B) compared with prior chest X-ray (A) Cardiomegaly is demonstrated by arrow in B

**Figure 2:**
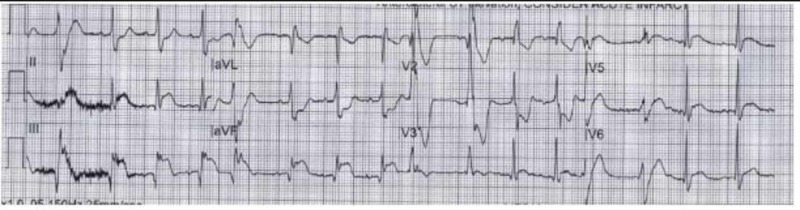
Initial EKG strip Inferior-posterior wall myocardial infarction with ST elevations in leads II, III, and aVF

He was hypertensive at 152/113 mmHg and tachycardic at 114 beats per minute upon presentation. There were no other abnormal findings on physical examination including cardiovascular exam; lipid panel was within normal limits and troponin I was elevated at 7.7 ng/mL (normal reference range 0.000-0.045 ng/mL). Chest X-ray demonstrated cardiomegaly (Figure 1), and EKG was remarkable for inferior-posterior wall ST elevation myocardial infarction (STEMI) as shown in Figure 2.

**Figure 3:**
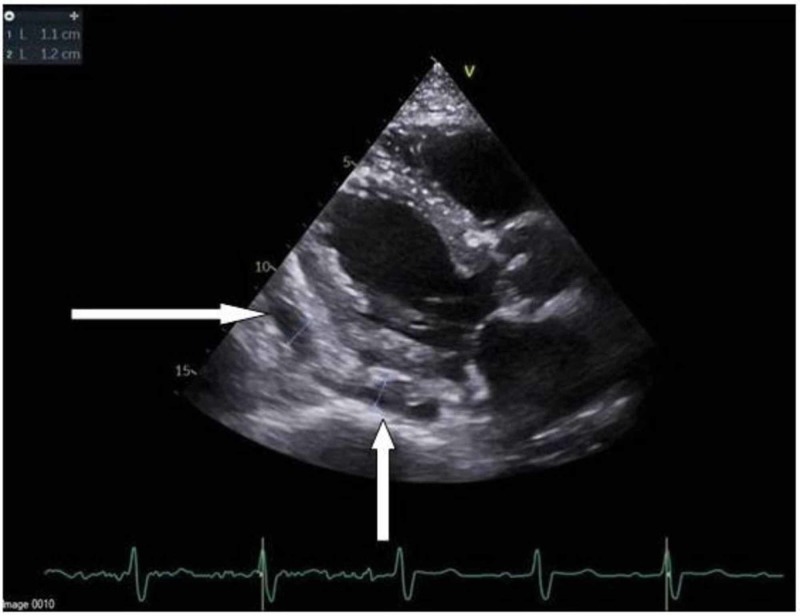
PLAX view of echocardiogram Thrombosed pseudoaneurysm demonstrated measurements in the top-left corner by arrows PLAX, parasternal long axis

**Figure 4:**
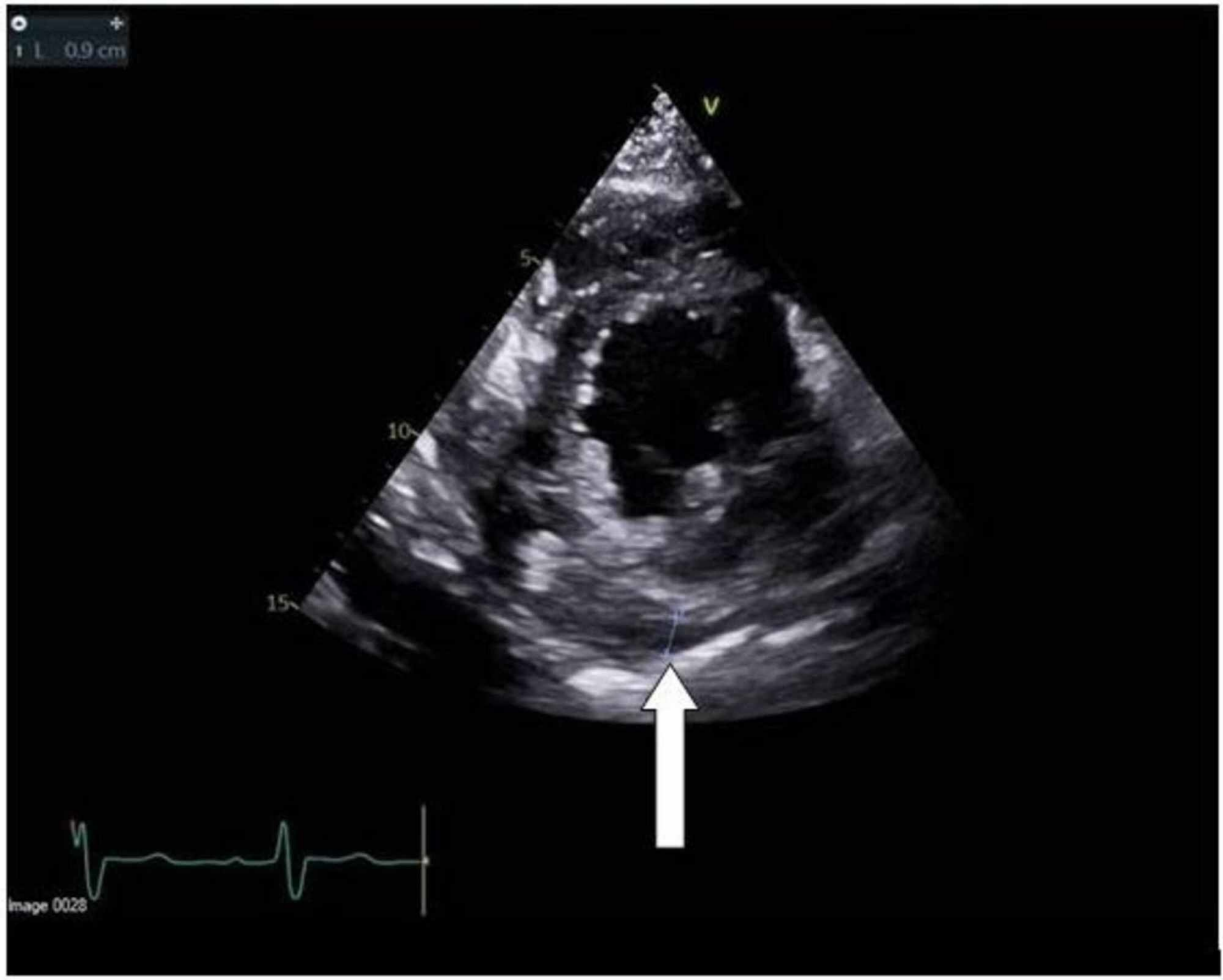
PSAX view of echocardiogram demonstrating thrombosed pseudoaneurysm PSAX: parasternal short axis

While awaiting early revascularization, 2D echocardiogram was performed and demonstrated moderately to severely reduced left ventricular systolic function, with an ejection fraction ranging between 30% and 35% with grade 1 diastolic dysfunction. Hypokinesis of the entire inferior, basal-mid inferolateral, basal-mid anterolateral, and apical lateral myocardium was noted, with an area of complex fluid collection within the pericardium, larger on the right side. A small area with no obvious active flow within the mid to basal inferior wall was suggestive of a thrombosed pseudoaneurysm (Figures 3, 4).

We performed early revascularization; a completely occluded SVG to RCA with a thrombus was found, and the patient underwent stenting of SVG to 100% occluded RCA. There was also a completely occluded native left main coronary artery with patent vein graft to the OM artery, and patent vein graft to LAD. 

We consulted with our cardiothoracic surgery team and decided to observe this patient on optimal medical therapy. If he developed heart failure symptoms, valve repair and pseudoaneurysm repair would be considered at that point. He was discharged home on aspirin, atorvastatin, furosemide, losartan, spirinolactone, and ticagrelor, with plans to follow up in a month.

## Discussion

Ventricular pseudoaneurysms have become increasingly rare due to improved medical and interventional management of acute MI. Its incidence is reportedly as low as <0.3% [1,2]. When they occur, mortality can be as high as 40% with complications such as rupture and potentially fatal cardiac tamponade, thromboembolism, focus of infection, and arrhythmia [3]. However in about 10% of cases, patients present asymptomatically with later-found incidental findings [4]. It is known that predisposing factors to the development of left ventricular aneurysms include MI particularly of the inferior wall followed by anterior wall infarction, and cardiac surgery responsible for about 33% of cases [4]. Procedures such as mitral valve replacement and aneurysmectomy account for a larger percentage of cases. There is limited data for left ventricular pseudoaneurysm incidence after CABG as there are only a few reported cases. Our patient had undergone three-vessel CABG about 10 years prior to presentation. Traumatic left ventricular pseudoaneurysms have also been reported [5]. Whereas a true aneurysm can usually be managed medically, false or pseudoaneurysms usually require surgical treatment [6]. Current data do not support prophylactic aneurysmectomy of stable chronic pseudoaneurysms [7].

Symptomatic patients will often present with symptoms of coronary artery disease or congestive heart failure with shortness of breath or chest pain like our patient. When asymptomatic, pseudoaneurysms are often detected by echocardiography, which is more commonly used in clinical practice [3,4,6]. Other diagnostic modalities include cardiac MRI, CT, and ventriculogram. The pseudoaneurysm will usually have bidirectional blood flow into its cavity but in our described case, there was thrombosis of the false aneurysm without blood flow. Our patient’s presentation with a pseudoaneurysm in the distribution of the occluded SVG makes it likely that it was a complication of current inferior-posterior wall STEMI. The finding of a complex fluid collection in the pericardium was consistent with myocardial rupture with subsequent organized thrombus in the pericardial space creating a false aneurysm. Metastatic renal cell carcinoma to the heart was considered as a possible etiology of pericardial effusion due to his prior history of renal cell carcinoma; however, pseudoaneurysm was more likely given his presentation with an inferior-posterior wall STEMI.

Traditionally, surgical management is favored over medical management given the high risk of complications including rupture despite surgery-associated mortality risks [4]. Due to the recent MI with necrotic tissue and the hemodynamic stability of this patient, the patient was discharged home on optimal medical therapy with plans to follow up for a repeat echocardiogram in a month, and for considerations for valve and pseudoaneurysm repair if symptoms of heart failure develop.

## Conclusions

Pseudoaneurysms are rare complications after MIs due to current aggressive angiography and catheterization. Given our patient’s medical noncompliance which favored development of cardiovascular disease and complications, our reported patient presented 10 years after CABG with an inferior-posterior wall STEMI complicated by a thrombosed pseudoaneurysm. Medical management was opted for due to the pseudoaneurysm presentation with plans to consider surgery if symptoms of heart failure and/or hemodynamic compromise develop. His chest pain was completely resolved, and he was hemodynamically stable prior to discharge.
